# A preliminary analysis of hepatitis C virus in pancreatic islet cells

**DOI:** 10.1186/s12985-017-0905-3

**Published:** 2017-12-20

**Authors:** Jason T. Blackard, Ling Kong, Angela Lombardi, Dirk Homann, Sara Salehi Hammerstad, Yaron Tomer

**Affiliations:** 1 Division of Digestive Diseases, Department of Internal Medicine, University of Cincinnati College of Medicine, ML 0595, 231 Albert Sabin Way, Cincinnati, OH 45267 USA; 2 Department of Medicine, Albert Einstein College of Medicine and Montefiore Medical Center, Bronx, NY USA; 3 Diabetes Obesity and Metabolism Institute, Mount Sinai Medical Center, New York, NY USA; 4 Department of Pediatrics, Oslo University Hospital, Oslo, Ullevål Norway

**Keywords:** Hepatitis C virus, Pancreas, Islet cells, Extrahepatic replication, Diabetes

## Abstract

**Background:**

An association between hepatitis C virus (HCV) and type 2 diabetes (T2D) is supported by numerous epidemiologic studies. We hypothesized that HCV could infect human pancreatic islet cells in vitro.

**Methods:**

Measures of HCV RNA synthesis and protein production were used to evaluate HCV infection of pancreatic islets recovered from human donors.

**Results:**

Significant co-staining of insulin and the HCV entry factor CD81 was observed in pancreatic islets. Positive- and negative-sense HCV RNA were detected in HCV-exposed islets at days 1, 3, 7, and 14 post-infection. The HCV core and NS3 proteins were expressed and increased with time providing further evidence of viral replication. Interferon and an HCV polymerase inhibitor reduced viral replication in islet cells. In HCV-infected islets, TNFα levels were elevated at days 1, 3, and 7 post-infection, while IL-6 levels were elevated at day 1 but not days 3 or 7. Overall, the expression of miR-122 was low in islets compared to the Huh7.5 hepatocyte-derived cell line, although the relative expression of miR-122 increased in islet cells after viral infection (1, 6.63, and 5.83 at days 1, 3, and 7, respectively).

**Conclusions:**

In this pilot study, viral infection was demonstrated in pancreatic islet cells from multiple donors using complementary measures of viral replication, thus providing evidence of in vitro infection. Altered cytokine expression may contribute to the development of insulin deficiency, and understanding the etiology of diabetes in individuals with HCV infection may facilitate the development of novel treatment modalities and prevention strategies. This in vitro system provides an important model for mechanistic studies of HCV-pancreas interactions and facilitates future studies of the potential impact of viral infection on islet cell function.

## Background

Over 1.9 billion adults worldwide are overweight and **>**600 million are obese, corresponding to 39% and 13 of world’s adult population, respectively [1]. Multiple genetic and dietary factors are associated with the development of type 2 diabetes (T2D), although less is know about the role of certain environmental factors such as viral infection. Globally, 130–170 million people are infected with hepatitis C virus (HCV) [2]. While hepatocytes represent the major site of viral replication, liver disease is not the sole outcome of HCV replication, and extrahepatic complications of HCV infection are common and complicate its management (reviewed in [3]). HCV infection and interferon-based therapies frequently induce endocrine-metabolic complications, including diabetes [4, 5]. Indeed, an association between chronic HCV infection and T2D and insulin resistance has been demonstrated consistently (reviewed in [6]), as well as an increased risk of pancreatic cancer [7, 8].

Multiple studies have demonstrated HCV replication in several extrahepatic tissues and cell types, suggesting that at least some of the extrahepatic manifestations may be caused directly by the virus (reviewed in [9]). HCV RNA has been detected in the pancreata of patients with chronic HCV suggesting that viral infection occurs in vivo [10–12]. For instance, Laskus et al. detected negative-sense HCV RNA – a replication intermediate – in 5 of 8 post-mortem pancreatic tissues [10]. Similarly, Yan et al. detected negative-sense HCV RNA and/or viral antigens in pancreata from multiple individuals [11]. Virus-like particles have also been observed in pancreatic beta cells from individuals with HCV infection [13]. Preliminary data suggest that HCV sequences in the pancreas are distinct from those circulating in the periphery, offering further evidence of viral adaptation for efficient replication within the pancreas [12]. Nonetheless, the particular cell types supporting viral replication in the pancreas have not been evaluated extensively, and in vitro models to examine the impact of viral infection on pancreatic cell function are limited.

This pilot study provides preliminary evidence of viral infection of pancreatic islet cells in vitro. This system will be valuable for future studies that further characterize the viral and host factors that facilitate HCV infection of the pancreas and for exploring the mechanisms by which HCV infection may promote the development of diabetes and insulin resistance.

## Methods

### Cell culture

Human hepatocyte (Huh7.5) and human embryonic kidney (293 T) cell lines were provided by Apath LLC (St. Louis, MO) and maintained in Dulbecco’s Modified Eagle’s Medium (DMEM) high glucose supplemented with 10% fetal bovine serum (FBS), penicillin (100 U/mL), streptomycin (100 mg/mL), and non-essential amino acids.

All human samples (islets) were received from the Integrated Islet Distribution Program (IIDP) [14] and were de-identified / anonymous to the study investigators. The study was reviewed and approved by the Icahn School of Medicine Institutional Review Board as exempt (GCO#: 09–1593). Donors had no evidence of type 1 or type 2 diabetes. Islets were maintained in RPMI 1640 supplemented with glucose 5.5 mM and 10% FBS. Islets and Huh7.5 cells were cultured at 37 °C in 5% CO_2_, and medium was replaced every 2–4 days.

### mRNA expression of HCV entry factors

Total RNA from 3 different human islets donors was isolated using TRIzol reagent (Thermo Scientific) in combination with the RNeasy Mini kit (Qiagen) followed by DNase treatment. Five hundred nanograms of total RNA were retrotranscribed using the Superscript III kit (Thermo Scientific), and the cDNAs obtained were utilized as templates for quantitative real-time RT-PCR analysis of CD81 (72 base pair [bp]), occludin (63 bp), claudin-1 (101 bp), and SR-B1 (50 bp), as well as the housekeeping gene GAPDH (94 bp). cDNA was run on an AbiPRISM 7300 fast real-time cycler using the power SYBR Green real-time PCR master mix kit and quantified by built-in SYBR Green Analysis. All samples were evaluated in triplicate with the average relative amount of specific mRNA being normalized to glyceraldehyde 3-phosphate dehydrogenase (*GAPDH*) expression. The mRNA levels in Huh7.5 cells and human islets are shown relative to those in 293 T cells.

### CD81 staining of islet cells

Islet cells were cultured overnight and dispersed using standard enzymatic or non-enzymatic protocols and stained for viability. Surface CD81 or IgG_1_ isotype and intracellular insulin expression were evaluated by flow cytometry. Plots were gated on live cells with high FSC/SSC properties typical of endocrine cells.

### Production of infectious HCV particles and infection of islet cells

The Huh7.5_JFH1_ cell line – which releases infectious HCV genotype 2a virions into the cell culture supernatant – was provided by Dr. Guangxiang Luo [15] and maintained in DMEM with 10% FBS and 5 μg/mL of blasticidin. Infectious virions (hereafter referred to as JFH1) were harvested from the supernatants of Huh7.5_JFH1_ cells, filtered, spun at high speed to pellet cellular debris, and stored at −80 °C prior to use. For all experiments, 1 × 10^5^ islet cells or Huh7.5 cells were infected for 4 h with 0.5 TCID_50_ of virus in a 24-well plate. Virus was then removed, and cells were washed with PBS multiple times to remove unbound virus. Given the limited availability of patient-derived islet cells, each experiment was performed using islet preparations from at least 2 donors. All results shown reflect representative experiments with error bars showing replicate infections within the same islet cell preparation.

### Qualitative strand-specific reverse transcription (RT)-PCR

RNA from cell lysates was extracted using TRIzol (Invitrogen; Carlsbad, CA), washed, and resuspended in 50 uL of DEPC-treated dH_2_O. RNA from 140 uL of culture supernatant was extracted using the QIAamp Viral RNA Kit (Qiagen; Valencia, CA), and eluted in 60 uL of DEPC-treated dH_2_O. HCV RNA was detected utilizing two qualitative strand-specific RT-PCR assays as described previously [16, 17]. PCR primers included the HCV-II sense primer (5’-CAC TCC CCT GTG AGG AAC T-3′, nucleotides [nt] 38–56 of the 5′UTR) and the HCV-I antisense primer (5′-TGG ATG CAC GGT CTA CGA GAC CTC-3′, nt 342–320) or the antisense primer KY78 (5’-CTC GCA AGC ACC CTA TCA GGC AGT-3′, nt 311–288) and sense primer KY80 (5’-GCA GAA AGC GTC TAG CCA TGG CGT-3′, nt 68–91). 30 cycles of PCR (94 °C for 30 s, 58 °C for 1 min, and 72 °C for 2 min) were performed, and PCR products (295 base pairs in length for HCV-I/-II and 244 base pairs for KY78/80) were visualized by gel electrophoresis.

### ELISA for HCV proteins

HCV core protein was quantified in cell culture supernatants by the QuikTiter HCV Core Antigen ELISA Kit (Cell Biolabs, Inc.; San Diego, CA), while HCV NS3 was quantified in cell lysates using the Quantitative HCV NS3 ELISA Kit (BioFront Technologies Inc.; Tallahassee, FL). Protein levels were compared to a standard curve, and both assays have lower limits of detection of 1 ng/mL.

### Inhibition of HCV replication

To evaluate cellular factors involved in viral entry, anti-CD81 monoclonal antibody (MA1–80820; Thermo Scientific) or IgG isotype control antibody (A3812; Sigma-Aldrich) were incubated with islets or Huh7.5 cells 1 day prior to and during HCV infection at dilutions of 1:100 and 1:2500. Incubation with 0.1 ng or 1000 ng of consensus interferon (Infergen**®** from Three Rivers Pharmaceuticals, LLC; Cranberry Township, PA) was performed 1 day before and during viral infection. A single dose of sofosbuvir (Gilead) was added at a concentration of 0.25 mg/mL.

### Cytokine expression

Cells were infected as described above, and culture supernatants were collected at days 1, 3, and 7 post-infection. IL-6, IL-8, TNFα, IL-1β, IL-12(p40), IL-17, and IFNα were measured using the Luminex multiplex assay (EMD Millipore Co; Bilerica, MA) with a lower limit of detection of 3.2 pmol/mL.

### MicroRNA-122 (miR-122) expression

Total RNA was extracted from islets and Huh7.5 cells as described above. Reverse transcription was performed with 40 ng of total RNA using the TaqMan microRNA reverse transcription kit (Applied Biosystems; Foster City, CA), a specific RT primer for miR-122 (UGG AGU GUG ACA AUG GUG UUU G), and the endogenous control miR-16 (UAG CAG CAC GUA AAU AUU GGC G). Therefore, only the miRNAs of interest were reverse transcribed into cDNA. cDNA was amplified using miRNA-specific PCR primers provided in the TaqMan microRNA assay and the TaqMan Universal PCR master mix with uracil N-glycosylase. Results were quantified using the Applied Biosystems 7300 Real-Time PCR system and expressed using the ΔΔCt method.

### Statistical analysis

Data are expressed as mean + SEM. Means were tested for statistical significance using the Student’s t-test. A significance level of *p* < 0.05 was applied when comparing virus-treated with untreated cells. Statistical analyses were performed using GraphPad Prism 5 (San Diego, CA).

## Results

### Human islets express HCV entry factors

**Fig. 1:**
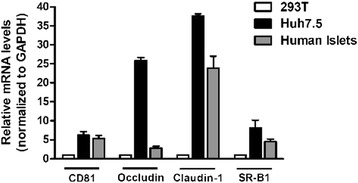
Total RNA from 3 different human islets donors was isolated using TRIzol reagent in combination with the RNeasy Mini kit followed by DNase treatment. 500 ng of total RNA were retrotranscribed using the Superscript III kit. The cDNAs obtained after retrotranscription were used as templates for quantitative real-time RT-PCR for mRNAs corresponding to the HCV entry factors CD81, occludin, claudin-1, and SR-B1. The relative amount of specific mRNA was normalized to Glyceraldehyde 3-phosphate dehydrogenase (*GAPDH*). mRNA levels in Huh7.5 cells and human islets are relative to those in 293 T cells. Bars represent means ± SEM from three independent experiments

**Fig. 2:**
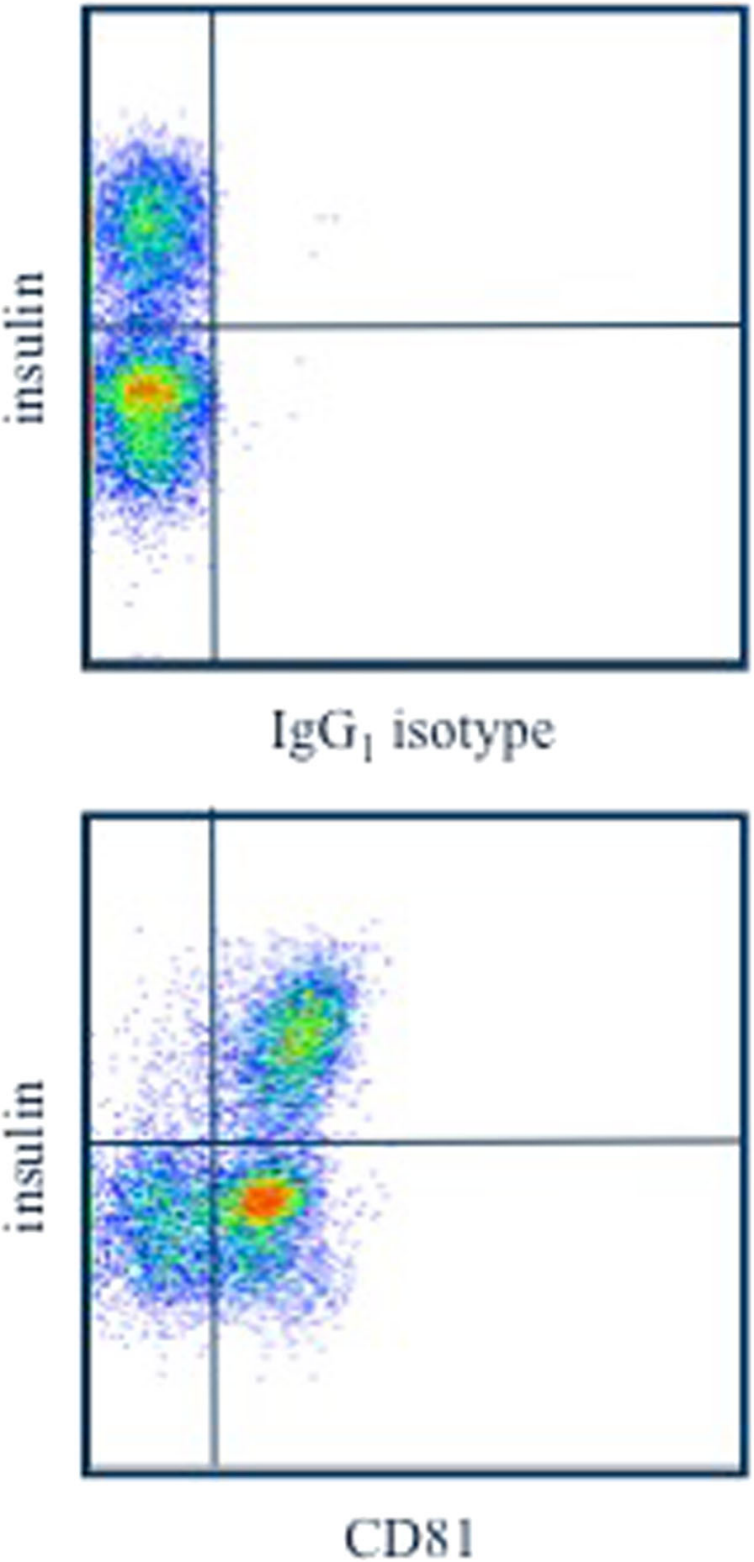
Islet cells were cultured overnight and then dispersed enzymatically. Cells were then stained for viability, surface CD81 (or IgG_1_ isotype), and intracellular insulin. Dot plots are gated on live cells with high FSC/SSC properties typical of endocrine cells. Insulin negative cells are mostly alpha cells

Several molecules have been identified as HCV attachment/entry factors in hepatocytes, including CD81 and may serve as future targets for antiviral drug design (reviewed in [18, 19]); nonetheless, their characterization in extrahepatic tissues and cell types is not well defined. As shown in Fig. 1, mean CD81 expression was 6.00 in Huh7.5 cells and 5.47 in 3 islet donors relative to expression in the 293 T cell line. Relative occludin levels were 26.13 and 2.90 in Huh7.5 cells and islet donors, respectively. Claudin-1 levels were 36.93 and 22.87, and SR-B1 levels 8.03 and 4.67. In Fig. 2, surface CD81 and intracellular insulin co-expression were evaluated in human islets after enzymatic dispersion. Significant co-staining of insulin and CD81 was observed, suggesting that beta cells express the CD81 protein.

### HCV can infect pancreatic islet cells

**Fig. 3:**
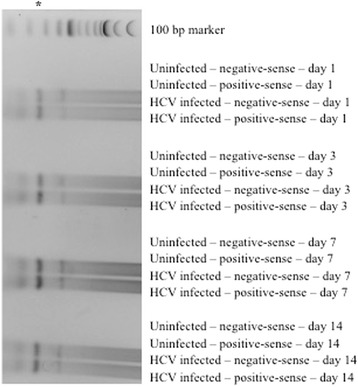
Qualitative reverse transcriptase PCR for the detection of positive-sense and negative-sense HCV RNA at days 1, 3, 7, and 14 post-infection of islet cell preparation #1; asterisks denote the 295 bp PCR product

Because of the strong epidemiologic association between HCV infection and diabetes, we evaluated if human islet cells could be infected productively with HCV in vitro. Hepatitis C virions contain positive-sense RNA genomes; thus, the detection of positive-sense HCV RNA is not sufficient to demonstrate replication within a particular cell type. Rather, evaluation of negative-sense HCV RNA – a replicative intermediate that is absent from virions and present only in productively infected cells – is crucial. Using a highly sensitive, qualitative RT-PCR, both positive- and negative-sense HCV RNA were evaluated in the cell lysates from pancreatic islet cells and the Huh7.5 hepatocyte-derived cell line after incubation with JFH1. Both positive-sense and negative-sense HCV RNA were detected in islet cell lysates at days 1, 3, 7, and 14 post-infection (Fig. 3), suggesting the occurrence of productive infection. As expected, positive-sense and negative-sense HCV RNA were not detected in cell lysates from uninfected pancreatic islet cells at any time point post-infection (Figs. 3 and 6). As well, HCV RNA was detected in Huh7.5 cells after incubation with JFH1 (data not shown).

**Fig. 4:**
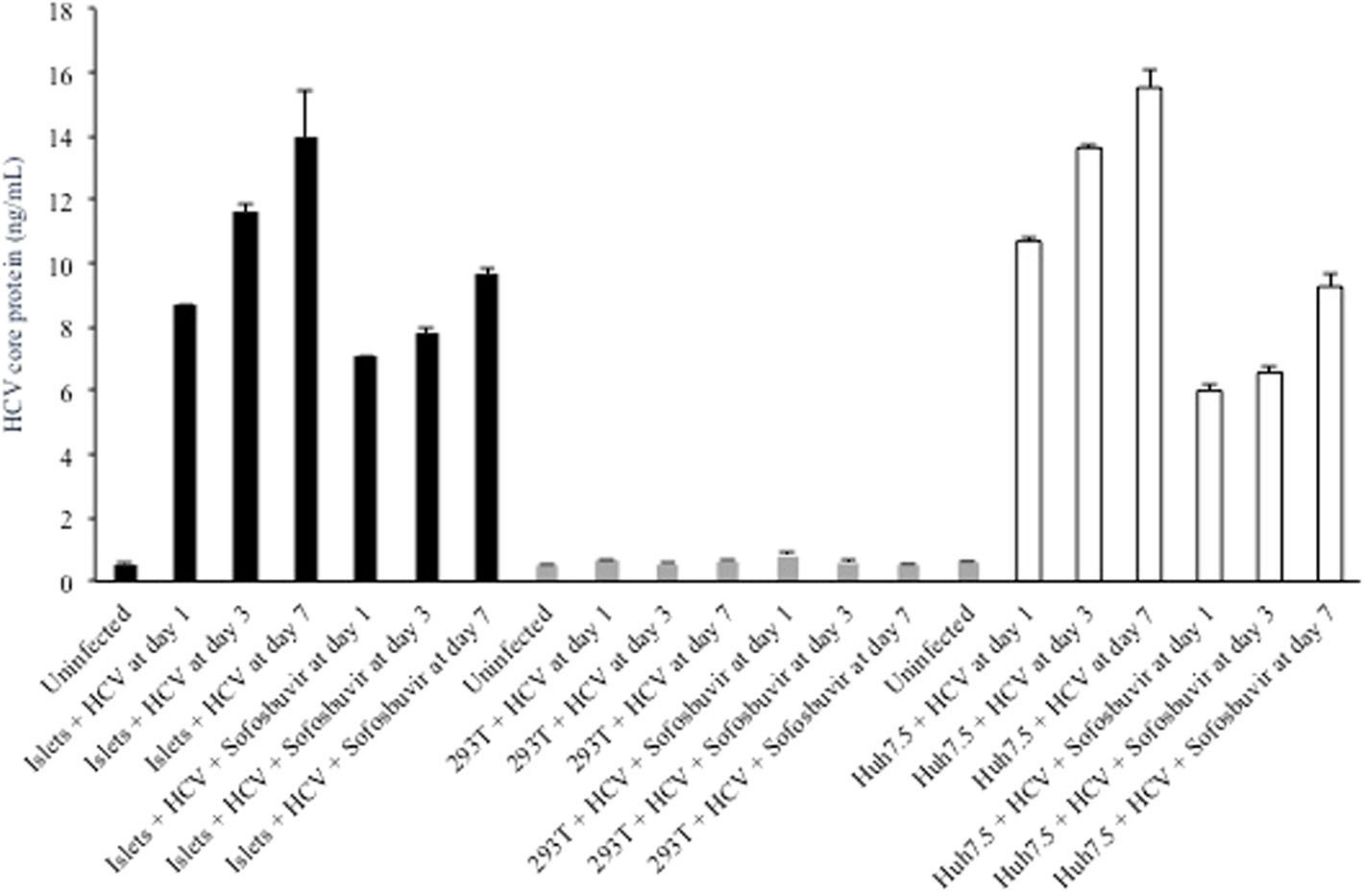
Supernatant HCV core protein levels (ng/mL) at days 1, 3, and 7 post-infection of Huh7.5 (white bars), 293 T (grey bars), or islet cell preparation #4 (black bars) in the presence or absence of sofosbuvir

HCV-infected pancreatic islet cells were further evaluated for viral protein production. As shown in Fig. 4, HCV core protein was expressed in islet cell supernatants at days 1, 3, 7, and 14 post-infection with JFH1 (compare levels of core protein in the “uninfected” condition to core protein levels at day 1, 3, and 7 post-infection). Similar results were obtained in additional islet cell donors, although absolute levels of infection were donor-dependent (data not shown). HCV core levels were higher in the Huh7.5 hepatocyte derived cell line. As an additional control, HCV core levels were evaluated in the non-permissive human kidney 293 T cell line; no significant core protein expression was detected above background levels.

**Fig. 5:**
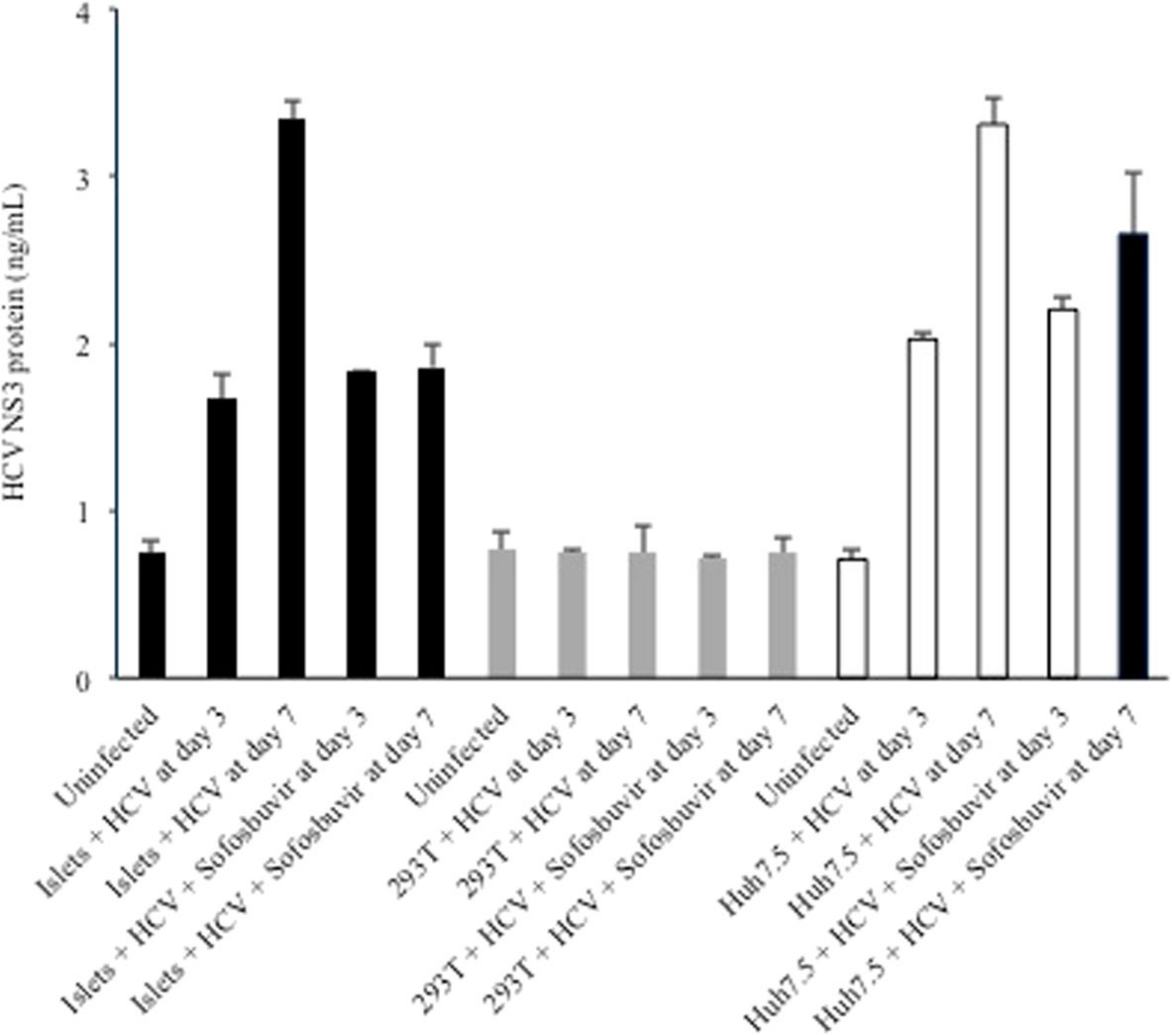
Cell lysate levels of HCV NS3 protein (ng/mL) at days 3 and 7 post-infection of Huh7.5 (white bars), 293 T (grey bars), or islet cell preparation #4 (black bars) in the presence or absence of sofosbuvir

Because HCV core protein is contained with infectious virions, initial findings were confirmed by detection of the HCV NS3 protein, which is not present in virions but required for genome replication. As shown in Fig. 5, HCV NS3 protein was increased in cell lysates at days 3 and 7 post-infection compared to uninfected cells providing further evidence of bona fide infection.

### The HCV polymerase inhibitor sofosbuvir and IFNα limit HCV replication in pancreatic islet cells

**Fig. 6:**
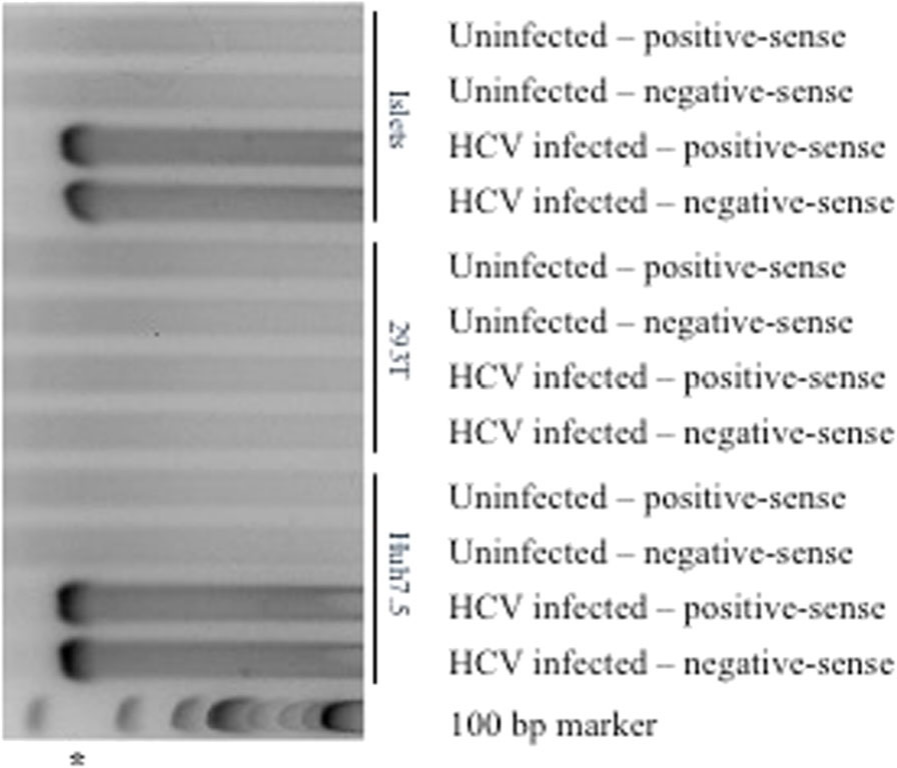
Qualitative reverse transcriptase PCR for the detection of positive-sense and negative-sense HCV RNA at day 1 post-infection of islet cell preparation #4 (top), 293 T (middle), and Huh7.5 (bottom); asterisk denotes the 244 bp PCR product

Sofosbuvir is a nucleotide analog inhibitor of the HCV RNA-dependent RNA polymerase (NS5B) that is currently approved for the treatment of chronic HCV infection. At a concentration of 0.25 mg/mL, sofosbuvir treatment of HCV-infected cells resulted in partial inhibition of viral replication in islet cells, as well as in Huh7.5 cells (Fig. 4). As expected, sofosbuvir had no impact on viral replication in 293 T cells, consistent with a previous report [20]. NS3 protein levels were reduced significantly in the presence of sofosbuvir as would be expected (Fig. 5). The presence of positive- and negative-sense HCV RNA was confirmed using a second qualitative, strand-specific RT-PCR assay in islets and Huh7.5 cells – but not 293 T cells – as shown in Fig. 6.

**Fig. 7:**
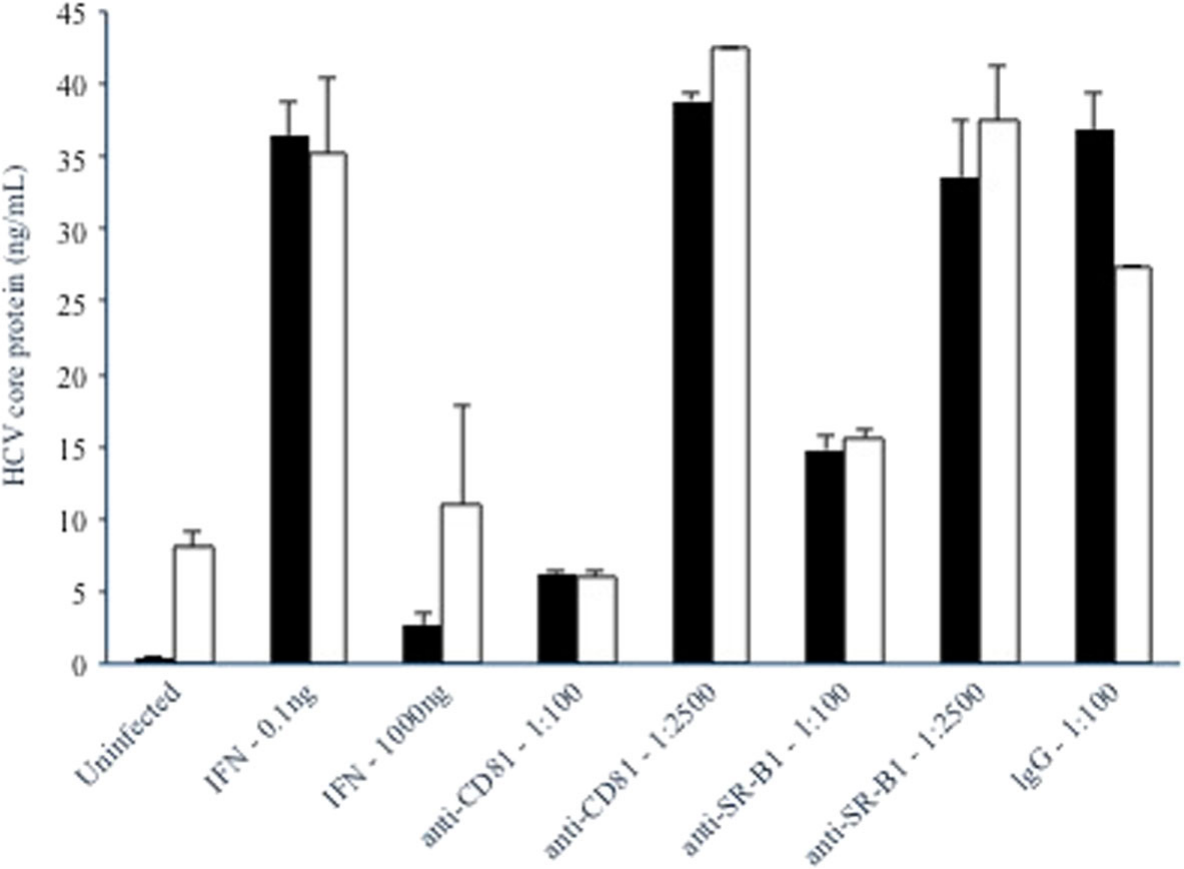
Supernatant HCV core protein levels (ng/mL) at day 3 post-infection of Huh7.5 (white bars) or islet cell preparation #5 (black bars) in the presence or absence of IFNα, anti-CD81, anti-SR-B1, or IgG isotype control antibodies

IFNα is a potent inhibitor of HCV replication in hepatocytes; therefore, its ability to inhibit JFH1 replication in pancreatic islet cells was evaluated. As shown in Fig. 7, a high dose (1000 ng) of IFNα inhibited JFH1 replication in pancreatic islet cells, while a low dose (0.1 ng) had not inhibitory effect.

### HCV infection of pancreatic islet cells is dependent on CD81 and SR-B1

To further investigate the role of HCV entry factors, we evaluated whether infection of pancreatic islet cells was dependent on CD81 and/or SR-B1. As shown in Fig. 7, anti-CD81 antibody was efficient at inhibiting JFH1 infection of pancreatic islet cells at a 1:00 dilution, although a 1:2500 dilution had no inhibitory effect compared to the IgG control condition. As expected, anti-CD81 antibody also efficiently inhibited JFH1 infection of Huh7.5 cells. Anti-SR-B1 antibody also inhibited JFH1 infection of pancreatic islet cells at the 1:00 dilution but had no effected at the 1:2500 dilution. Anti-SRB1 antibody also inhibited JFH1 infection of Huh7.5 cells. We have not yet evaluated the impact of antibodies against other HCV entry factors on viral infection of pancreatic cells, although this is an important line of investigation for future studies.

### HCV infection of pancreatic islet cells produces infectious virions

**Fig. 8:**
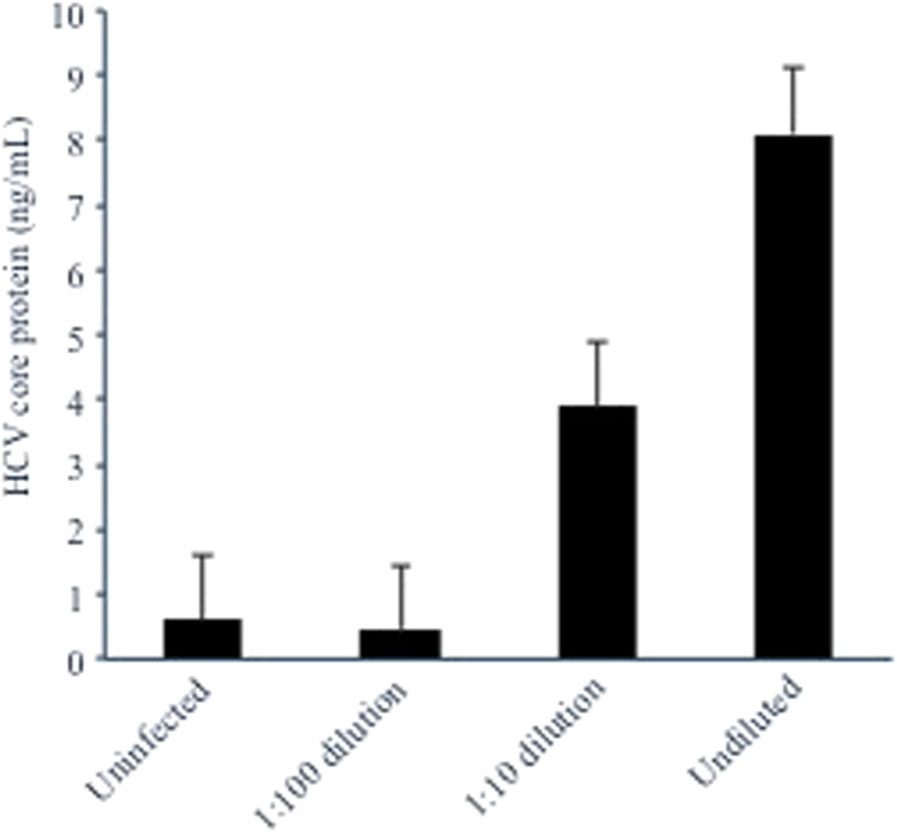
Islet cell preparation #3 was infected with HCV for 4 h, supernatants harvested at day 3 post-infection, and used to infect fresh Huh7.5 cells. Supernatant HCV core protein levels (ng/mL) were measured at day 3 post-infection to assess the production of infectious virions

Islet cells infected with JFH1 were evaluated for possible production of infectious virions capable of subsequent rounds of infection. Islets were first infected with JFH1 for 4 h as in other experiments, and cells were washed with PBS multiple times to remove unbound virus. Supernatants were then collected at day 3 post-infection and used to infect naïve Huh7.5 cells. As shown in Fig. 8, a modest level of HCV infection was achieved and was concentration dependent, suggesting that islet cells are capable of producing infectious virions that can infect other cell types.

### HCV infection of pancreatic islet cells alters cytokine expression

**Fig. 9:**
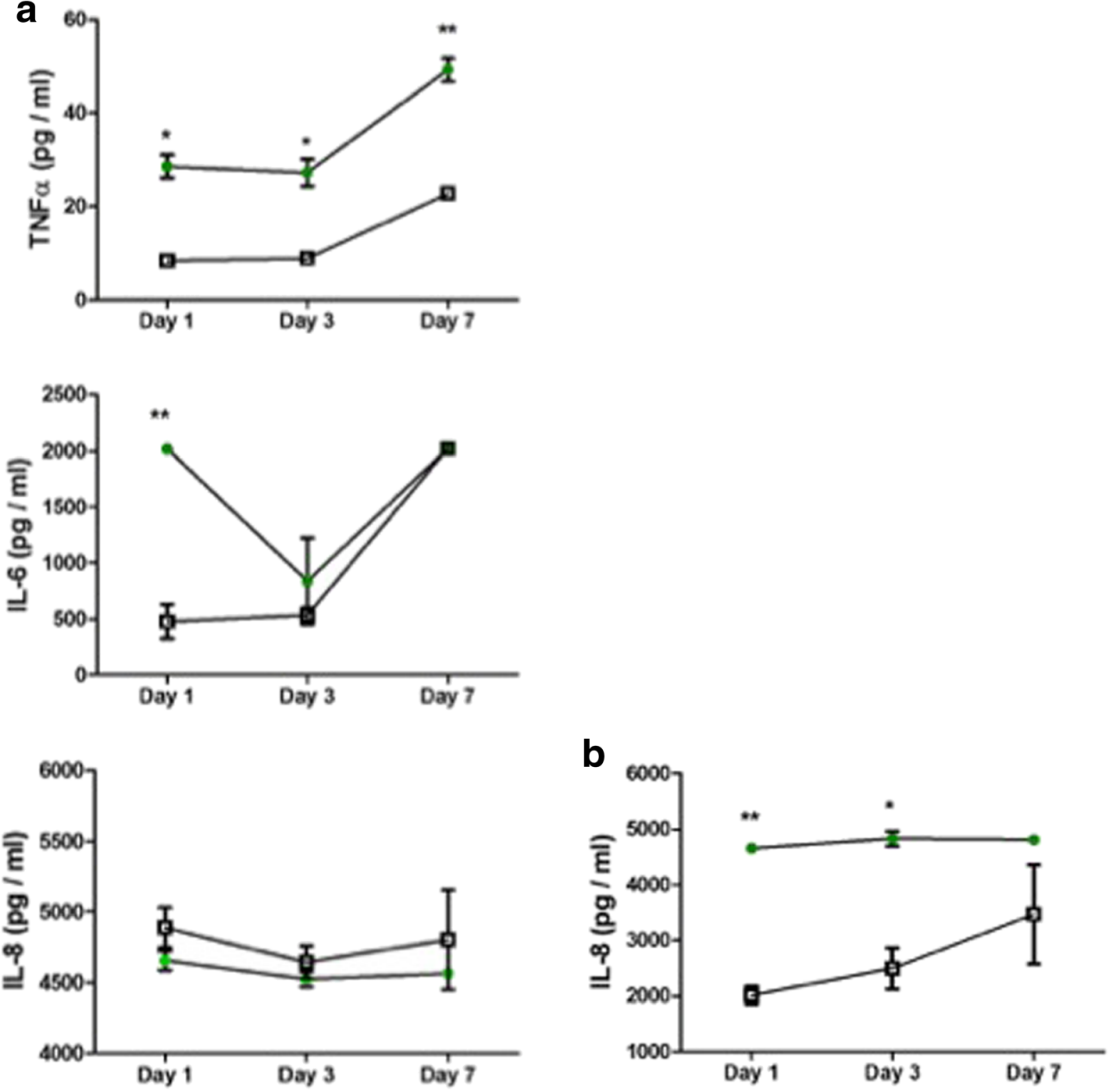
**a** Supernatant expression of IL-6, IL-8, and TNFα cytokines (pg/mL) at days 1, 3, and 7 post-infection of islet cell preparation #5 (closed circles) compared to control levels in uninfected islet cells (open squares). **b** Supernatant expression of IL-8 cytokines (pg/mL) at days 1, 3, and 7 post-infection of Huh7.5 cells (closed circles) compared to control levels in uninfected Huh7.5 cells (open squares). ** *p* < 0.01; * *p* < 0.05 for comparison of means using the Student’s t test

We have previously reported that the HCV E2 protein induces IL-8 expression in thyrocytes [21]. Therefore, we examined the impact of HCV infection on pro-inflammatory cytokine expression in islet cells. Compared to mock infected islet cells, cell culture supernatant levels of TNFα were elevated at days 1, 3, and 7 post-infection (Fig. 9a). IL-6 levels were elevated at day 1 but not day 3 or 7. IL-8 levels were not significantly different between mock infected and HCV-infected islet cells, although HCV infection induced increased production of IL-8 in Huh7.5 cells (Fig. 9b). Furthermore, no increase in the levels of IL-1β, IL-12(p40), IL-17, and IFNα was observed in infected islets or Huh7.5 compared to uninfected cells (data not shown).

### miR-122 is present in pancreatic islets and is upregulated after viral infection

miR-122 regulates HCV RNA abundance and is important for efficient production of infectious virus in hepatocytes [22–24]. Overall, the expression of miR-122 was low in islets compared to the Huh7.5 hepatocyte-derived cell line as indicated by high Ct values (36 versus 21 in islets and Huh7.5, respectively). Although the relative expression of miR-122 increased in islet cells after viral infection (1, 6.63, and 5.83 at days 1, 3, and 7, respectively), the expression remained low compared to uninfected or HCV-infected Huh7.5 cells. Thus, miR-122 is detectable in islet cells even though its exact role in this extrahepatic cell type has not been rigorously evaluated to date.

## Discussion

While significant advances have been made in the treatment of HCV in recent years, direct-acting agents are costly and not available to many individuals. Moreover, the focus on liver disease as the sole outcome of HCV replication is limited in scope and neglects the extrahepatic complications of this systemic infection. A large number of studies confirm an increased risk for T2D in patients with chronic HCV infection (reviewed in [6]). It is estimated that ~30% of patients with liver cirrhosis develop diabetes [25]. A meta-analysis of 34 studies reported a significantly higher risk of T2D in patients with HCV compared to patients with hepatitis B virus, matched controls, or patients with other forms of liver disease [26].

While HCV is hepatotropic, evidence of extrahepatic replication has been identified in a variety of non-hepatic tissues, including the thyroid, bone marrow, adrenal gland, spleen, lymph node, cervicovaginal fluid, and brain [10–12, 27–31]. Moreover, HCV RNA, viral antigens, and/or virus-like particles have been detected in the pancreata of patients with chronic HCV, highly suggestive of viral infection in vivo [10, 11, 13]. Nonetheless, direct evidence of viral replication in islets is lacking because of the difficulty in procuring the appropriate clinical samples. Wang et al. previously reported low-level HCV replication within the insulin-producing beta cell line MIN6 [32], although that cell line was murine in origin, infectious progeny virions were not released, and the authors utilized a higher multiplicity of infection than in the current study with human islets. Thus, we provide the first evidence of direct viral infection of pancreatic islet cells in vitro. Notably, viral infection was demonstrated in islets from multiple donors.

In this pilot study, infection in pancreatic islets was demonstrated using multiple complementary assays, including qualitative strand-specific RT-PCR for HCV RNA and quantitative ELISA for two HCV proteins. HCV replication was evaluated further by demonstrating inhibition with anti-CD81 antibodies anti-SR-B1 antibodies, sofosbuvir – a potent HCV polymerase inhibitor – and the antiviral cytokine IFNα. Viral RNA and protein were absent in uninfected islets, and a non-permissive cell line (293 T) showed no evidence of viral RNA or protein after in vitro exposure. We have previously shown detection of the NS5A non-structural protein by Western Blot in an extrahepatic cell type [16]; however the number of islets available prevented a similar approach here. The production of infectious virions in islet cells that were capable of subsequent rounds of infection was also shown.

miR-122 is highly abundant in the human liver and represents a determinant of efficient HCV replication in hepatocytes. miR-122 also plays an important role in regulating lipid homeostasis [33]. While not absolutely required, exogenous expression of miR-122 enhances HCV replication in non-hepatic cells [34, 35]. Other have reported decreased miR-122 expression in pancreatic cancer compared to healthy tissues [36] and a positive association between miR-122 mRNA levels in islets and insulin mRNA biosynthesis [37]. Given the critical role of lipids in the HCV life cycle, miR-122 regulation may impact viral replication in extrahepatic cell types as well and deserves additional investigation. Moreover, direct comparison of HCV replication levels in islets and primary hepatocytes from the same donor have not been performed; thus, the relevant permissiveness of these two cells types to infection cannot be compared directly. Finally, JFH1 is a genotype 2a strain of HCV, and the ability of other HCV genotypes to replicate within islet cells has not been explored yet.

It is important to note that while all islet cell preparations from multiple donors could be infected with HCV, the level of infection achieved showed donor-dependent variability suggesting that cellular factors likely play a critical role in the amount of viral replication that occurs. This in vitro finding may also partially explain why only a subset of individuals with HCV infection develops diabetes and requires additional investigation. While infection appears to be CD81-dependent and SR-B1-dependent, the role of other HCV entry factors have not been evaluated in the pancreas to date and should be investigated carefully in future studies and may require evaluation of multiple donors since replication levels may be dependent on donor-specific levels of a particular entry factor or a combination of entry factors. While we did quantify the mRNA levels of several HCV receptors, protein levels remain to be evaluated in islets. Additionally, other than the NS5B inhibitor sofosbuvir, the role of other direct-acting agents on replication within the pancreas was not evaluated. Finally, the concentration of sofosbuvir used was relatively high, although it is unclear if a higher sofosbuvir dose would efficiently eliminate in virus replication in the pancreas.

The mechanisms by which HCV may promote T2D include impairment of the insulin signaling pathway by viral proteins and HCV-induced liver inflammation resulting in the release of pro-inflammatory cytokines and chemokines that interfere with insulin signaling [38]. HCV can have direct effects on insulin signaling. Additionally, TNFα may play a pivotal role in the association between HCV infection and diabetes (reviewed in [6]). Nonetheless, the mechanisms by which HCV contributes to T2D may be distinct from previously characterized mechanisms of type 1 diabetes or T2D, and previous studies did not evaluate functional interactions between HCV and islet cells. Interestingly, we found that HCV infection of islet cells triggered altered cell expression of pro-inflammatory cytokines suggesting a possible connection between viral replication and islet cell function, although this deserves additional investigation. This represents an important model for studies of HCV-islet interactions by closely mimicking the in vitro physiologic environment.

## Conclusions

These findings are preliminary in nature. Nonetheless, they imply that direct infection of beta cells by HCV may play a key role in the association between HCV and type 2 diabetes. However, it should be noted that the exact cell that is infected by HCV is not critical to our hypothesis. We believe that the virus infects beta cells based on their high expression levels of CD81, although other cellular factors should be evaluated rigorously in future studies. Regardless, even if the virus infects alpha and beta cells (or only alpha cells), the resulting inflammation and cytokine response may damage both alpha and beta cells resulting in islet dysfunction and diabetes. Nonetheless, this is a pilot study, and these preliminary results should be viewed with caution until confirmed by others using complementary methodologies. Understanding the etiology of diabetes in individuals with HCV infection may enable the development of novel treatment modalities and prevention strategies based on the specific viral mechanisms that trigger diabetes. These results support the hypothesis that the pancreas serves as an extrahepatic reservoir of replication and that HCV treatment with direct-acting agents may ameliorate virus-mediated diabetes.

## References

[1] Obesity and overweight [http://who.int/mediacentre/factsheets/fs311/en/].

[2] Alter M (2007). Epidemiology of hepatitis C virus infection. World J Gastroenterol.

[3] Sherman AC, Sherman K (2015). Extrahepatic manifestations of hepatitis C infection: navigating CHASM. Curr HIV/AIDS Rep.

[4] Russo MW, Fried M (2003). Side effects of therapy for chronic hepatitis C. Gastroenterology.

[5] Tomer Y (2010). Hepatitis C and interferon induced thyroiditis. J Autoimmun.

[6] Hammerstad SS, Grock SF, Lee HJ, Hasham A, Sundaram N, Tomer Y (2015). Diabetes and hepatitis C: a two-way association. Front Endocrinol.

[7] Allison RD, Tong X, Moorman AC, Ly KN, Rupp L, Xu F, Gordon SC, Holmberg SD, Investigators CHCSC (2015). Increased incidence of cancer and cancer-related mortality among persons with chronic hepatitis C infection, 2006-2010. J Hepatol.

[8] Fiorino S, Cuppini A, Castellani G, Bacchi-Reggiani ML, Jovine E (2013). HBV- and HCV-related infections and risk of pancreatic cancer. JOP.

[9] Blackard JT, Kemmer N, Sherman K (2006). Extrahepatic replication of HCV: insights into clinical manifestations and biological consequences. Hepatology.

[10] Laskus T, Radkowsk M, Wang LJ, Vargas H, Rakela J (1998). Search for hepatitis C virus extrahepatic replication sites in patients with acquired immunodeficiency syndrome: specific detection of negative-strand viral RNA in various tissues. Hepatology.

[11] Yan FM, Chen AS, Hao F, Zhao XP, Gu CH, Zhao LB, Yang DL, Hao L (2000). Hepatitis C virus may infect extrahepatic tissues in patients with hepatitis C. World J Gastroenterol.

[12] Laskus T, Radkowski M, Wang LJ, Jang SJ, Vargas H, Rakela J (1998). Hepatitis C virus quasispecies in patients infected with HIV-1: correlation with extrahepatic replication. Virology.

[13] Masini M, Campani D, Boggi U, Menicagli M, Funel N, Pollera M, Lupi R, Del Guerra S, Bugliani M, Torri S (2005). Hepatitis C virus infection and human pancreatic beta-cell dysfunction. Diabetes Care.

[14] Ricordi C, Lacy PE, Finke EH, Olack BJ, Scharp D (1988). Automatic method for isolation of human pancreatic islets. Diabetes.

[15] Cai Z, Zhang C, Chang KS, Jiang J, Ahn BC, Wakita T, Liang TJ, Luo G (2005). Robust production of infectious hepatitis C virus (HCV) from stably HCV cDNA-transfected human hepatoma cells. J Virol.

[16] Blackard JT, Kong L, Huber AK, Tomer Y (2013). Hepatitis C virus infection of a thyroid cell line: implications for pathogenesis of hepatitis C virus and thyroiditis. Thyroid.

[17] Hiasa Y, Blackard JT, Lin W, Kamegaya Y, Horiike N, Onji M, Schmidt EV, Chung R (2006). Cell-based models of sustained, interferon-sensitive hepatitis C virus genotype 1 replication. J Virol Methods.

[18] Meredith LW, Wilson GK, Fletcher NF, McKeating J (2012). Hepatitis C virus entry: beyond receptors. Rev Med Virol.

[19] Douam F, Lavillette D, Cosset F (2015). The mechanism of HCV entry into host cells. Prog Nucleic Acid Res Mol Biol.

[20] Da Costa D, Turek M, Felmlee DJ, Girardi E, Pfeffer S, Long G, Bartenschlager R, Zeisel MB, Baumert T (2012). Reconstitution of the entire hepatitis C virus life cycle in nonhepatic cells. J Virol.

[21] Akeno N, Blackard JT, Tomer Y (2008). HCV E2 protein binds directly to thyroid cells and induces IL-8 production: a new mechanism for HCV induced thyroid autoimmunity. J Autoimmun.

[22] Henke JI, Goergen D, Zheng J, Song Y, Schuttler CG, Fehr C, Junemann C, Niepmann M (2008). microRNA-122 stimulates translation of hepatitis C virus RNA. EMBO J.

[23] Jopling CL, Yi MK, Lancaster AM, Lemon SM, Sarnow P (2005). Modulation of hepatitis C virus RNA abundance by a liver-specfiic microRNA. Science.

[24] Jangra RK, Yi MK, Lemon S (2010). Regulation of hepatitis C virus translation and infectious virus production by the microRNA miR-122. J Virol.

[25] García-Compeán D, González-González JA, Lavalle-González FJ, González-Moreno EI, Villarreal-Pérez JZ, Maldonado-Garza H (2015). Current concepts in diabetes mellitus and chronic liver disease: clinical outcomes, hepatitis C virus association, and therapy. Dig Dis Sci.

[26] White DL, Ratziu V, El-Serag H (2008). Hepatitis C infection and risk of diabetes: a systematic review and meta-analysis. J Hepatol.

[27] Radkowski M, Wilkinson J, Nowicki M, Adair D, Vargas H, Ingui C, Rakela J, Laskus T (2002). Search for hepatitis C virus negative-strand RNA sequences and analysis of viral sequences in the central nervous system: evidence of replication. J Virol.

[28] Laskus T, Radkowski M, Piasek A, Nowicki M, Horban A, Cianciara J, Rakela J (2000). Hepatitis C virus in lymphoid cells of patients coinfected with human immunodeficiency virus type 1: evidence of active replication in monocytes/macrophages and lymphocytes. J Infect Dis.

[29] Laskus T, Radkowski M, Wang L, Vargas H, Rakela J (1998). The presence of active hepatitis C virus replication in lymphoid tissue in patients coinfected with human immunodeficiency virus type 1. J Infect Dis.

[30] Nowicki M, Laskus T, Nikolopoulou G, Radkowski M, Wilkinson J, Du WB, Rakela J, Kovacs A (2005). Presence of hepatitis C virus (HCV) RNA in the genital tracts of HCV/HIV-1-coinfected women. J Infect Dis.

[31] Radkowski M, Kubicka J, Kisiel E, Cianciara J, Nowicki M, Rakela J, Laskus T (2000). Detection of active hepatitis C virus and hepatitis G virus/GB virus C replication in bone marrow in human subjects. Blood.

[32] Wang Q, Chen J, Wang Y, Han X, Chen X (2012). Hepatitis C virus induced a novel apoptosis-like death of pancreatic beta cells through a caspase 3-dependent pathway. PLoS One.

[33] Fernández-Hernando C, Ramírez CM, Goedeke L, Suárez Y (2013). MicroRNAs in metabolic disease. Arterioscler Thromb Vasc Biol.

[34] Chang J, Guo JT, Jiang D, Guo H, Taylor JM, Block T (2008). Liver-specific microRNA miR-122 enhances the replication of hepatitis C virus in nonhepatic cells. J Virol.

[35] Lin LT, Noyce RS, Pham TN, Wilson JA, Sisson GR, Michalak TI, Mossman KL, Richardson C (2010). Replication of subgenomic hepatitis C virus replicons in mouse fibroblasts is facilitated by deletion of interferon regulatory factor 3 and expression of liver-specific microRNA 122. J Virol.

[36] Papaconstantinou IG, Manta A, Gazouli M, Lyberopoulou A, Lykoudis PM, Polymeneas G, Voros D (2013). Expression of microRNAs in patients with pancreatic cancer and its prognostic significance. Pancreas.

[37] Bolmeson C, Esguerra JL, Salehi A, Speidel D, Eliasson L, Cilio C (2011). Differences in islet-enriched miRNAs in healthy and glucose intolerant human subjects. Biochem Biophys Res Commun.

[38] Antonelli A, Ferrari SM, Giuggioli D, Di Domenicantonio A, Ruffilli I, Corrado A, Fabiani S, Marchi S, Ferri C, Ferrannini E, Fallahi P (2014). Hepatitis C virus infection and type 1 and type 2 diabetes mellitus. World J Diabetes.

